# Investigation of variables affecting the immunogenicity of blood group antigens using a calculation formula

**DOI:** 10.1038/s41598-023-36078-4

**Published:** 2023-05-30

**Authors:** Yousun Chung, Han Joo Kim, Hyungsuk Kim, Sang-Hyun Hwang, Heung-Bum Oh, Dae-Hyun Ko

**Affiliations:** 1 Department of Laboratory Medicine, Kangdong Sacred Heart Hospital, Seoul, Korea; 2 Department of Laboratory Medicine, Asan Medical Center, University of Ulsan College of Medicine, 88, Olympic-Ro 43-Gil, Songpa-Gu, Seoul, 05505 Korea; 3 Department of Laboratory Medicine, Seoul National University Hospital, Seoul, Korea

**Keywords:** Humoral immunity, Anaemia

## Abstract

Previous studies on the immunogenicity of blood group antigens have utilized a formula incorporating antigen frequencies and relative frequencies of unexpected antibodies to the corresponding antigens. This study was aimed at investigating other variables potentially affecting the estimation of immunogenicity using this formula. We examined the effect of multiple transfusions, as there are more chance for a recipient to receive repeated transfusions rather than only once; the effect of antigen density, which may vary depending on homozygote/heterozygote; and the effect of unreliability of the observed frequency of rare antibodies and antigens. For multiple transfusions, the expected antibody frequency increased as the number of transfusions increased. For antigen density, the immunogenicity was falsely low for the low-prevalence antigen, and this tendency intensified as the effect of antigen density increased. Expected antibody frequencies were significantly affected by the uncertainties caused by estimation of small numbers. This study showed that the effects of various factors on the immunogenicity of blood group antigens depended on the antigen frequency. Estimating the immunogenicity of blood group antigens requires acknowledging the diverse factors that can affect it and interpreting the findings with caution.

## Introduction

Alloimmunization may occur after blood transfusion and limit securing compatible blood units or cause hemolytic disease of the newborn in some cases^1,2^ Extended antigen-matched transfusions are performed to prevent alloimmunization in patients with conditions requiring chronic transfusion therapy, such as sickle cell disease and thalassemia^3^ When determining which antigen to include in extended matching, the antigen frequency, clinical significance of the alloantibody, and immunogenicity of the antigen are considered. Studies have reported the immunogenicity of blood group antigens but with inconsistent results^4–7^.

Immunogenicity is estimated based on the probability of antigen-negative recipients receiving transfusions of antigen-positive blood and alloantibody frequency in the population, with or without modifications^4–7^ However, this method has the inherent limitation of being a retrospective analysis. Therefore, the present study was aimed at investigating other variables potentially affecting the estimation of immunogenicity using a formula by incorporating the antigen/antibody frequency.

## Materials and methods

### Basic concepts and principles of immunogenicity estimation

According to the basic concepts and principles of immunogenicity estimation formulas, for a specific antigen x, where *p*_*x*_ is the proportion of the population positive for the antigen, *q*_*x*_ is the proportion of the population negative for the antigen, and *Im*_*x*_ is the probability of immunization after an antigen-negative person receives a transfusion of antigen-positive blood, the probability that anti-x, an antibody against the antigen, is observed in the population (*Ab*_*x*_) is the product of the probability that the recipient is negative for the antigen (*q*_*x*_), probability that the transfused blood is positive for the antigen (*p*_*x*_), and probability that antibodies will be produced after transfusion (*Im*_*x*_), as shown in the following formula:

1 $$ Ab_{x} = p_{x} \times q_{x} \times Im_{x} $$

As the frequency of antibodies against red blood cell (RBC) antigens in the population has been reported, the basic logic of immunogenicity estimation is to calculate *Im* for specific antigens as the relative or absolute value. After the concept was first introduced by Giblett et al., other studies used the same logic with minor modifications in the formula^4–7^.

### Theoretical effect of multiple transfusion events

In clinical settings, there is a higher chance for a patient to receive multiple transfusions rather than only one, thereby increasing the probability of multiple exposures to antigen-positive blood. In patients with conditions that require chronic transfusions, immunogenicity estimation formulas should account for multiple transfusion events.

The probability of non-alloimmunization after one event (1–*Ab*_*x*_) was estimated as follows (1):

2 $$ 1 - Ab_{x} = 1 - p_{x} \times q_{x} \times Im_{x} $$

Assuming that the probability of alloimmunization in all transfusion events is independent of one another, the probability of non-alloimmunization against antigen x after multiple (*n*) transfusion events is as follows:

3 $$ 1 - Ab_{xn} = \left( {1 - p_{x} \times q_{x} \times Im_{x} } \right)^{n} $$

Similarly, the probability of alloimmunization against antigen x after transfusion events was estimated. Differences in the probability estimated using this formula compared to that estimated using the basic formula (1) according to *p*_*x*_ were assessed (*Effect*_*n*_) as follows:

4 $$ Ab_{xn} = 1 - \left( {1 - p_{x} \times q_{x} \times Im_{x} } \right)^{n} $$

5 $$ Effect_{n} = \left( 4 \right) \div \left( 1 \right) = \frac{{1 - \left( {1 - p_{x} \times q_{x} \times Im_{x} } \right)^{n} }}{{p_{x} \times q_{x} \times Im_{x} }} $$

### Theoretical effect of antigen density

Antigen density can vary, which may impact immunogenicity. Clinically significant antigens, such as RhD, Kell, Duffy, and Kidd, exhibit varying antigen density due to dosage effects^8,9^ Differences in antigen density in donor blood may result in distinct immune responses in the recipient. In this study, we evaluated the theoretical effects of antigen density on antibody frequency.

In general, one or more positive alleles out of a pair of alleles lead to the phenotypic expression of the antigen. Therefore, when *a*_*x*_ and *b*_*x*_ are the ratios of positive/negative alleles for antigen x (*a*_*x*_ + *b*_*x*_ = 1), the probability of negativity for antigen x (*q*_*x*_) is the homozygote status for the negative allele. The relationship between the allele type and the phenotype can be expressed using the following formula:

6 $$ b_{x} = \sqrt {q_{x} } $$

7 $$ a_{x} = 1 - b_{x} = 1 - \sqrt {q_{x} } $$

For calculation, we simplified the antigen density as being homozygote or heterozygote. For antigen positivity, the recipients should be homozygous for *a*_*x*_ (*a*_*x*_^*2*^) or heterozygous for *a*_*x*_ and *b*_*x*_ (2 X *a*_*x*_ X *b*_*x*_). If the immunogenicity of the heterozygote cell is lower than that of the homozygote cell (*I*_*m*_ X *l*, 0 ≤ *l* ≤ 1), the parameter applied to formula (2) is shown below. The probability of non-alloimmunization after transfusion can be expressed as the sum of the (i) probability of the recipient being antigen-positive (*p*_*x*_); (ii) probability of the recipient being negative and receiving antigen-negative blood (*q*_*x*_^2^); (iii) probability of the recipient being negative and transfused with heterozygous-positive blood without being sensitized (*q*_*x*_ X 2*a*_*x*_*b*_*x*_ X [1–*l* X *Im*_*x*_]); and (iv) probability of the recipient being negative and transfused with homozygous-positive blood without being sensitized (*q*_*x*_ X *a*_*x*_^*2*^ X [1–*Im*_*x*_]).

8 $$ 1 - Ab_{xdose} = p_{x} + q_{x}^{2} + 2q_{x} a_{x} b_{x} \left( {1 - l \times Im} \right) + q_{x} a_{x}^{2} \left( {1 - Im} \right) $$

Using formulas (6) and (7), the following formula was obtained:

9 $$ Ab_{xdose} = 1 - p_{x} - q_{x}^{2} - 2 q_{x} \sqrt {q_{x} } \left( {1 - \sqrt {q_{x} } } \right)\left( {1 - l \times Im} \right) - q_{x} \left( {1 - \sqrt {q_{x} } } \right)^{2} \left( {1 - Im} \right) $$

To evaluate the effects of formula (9) in comparison with that of formula (1), the output value of formula (9) was divided by that of formula (1) (*Effect*_*xdose*_ = *Ab*_*xdose*_ / *Ab*_*x*_).

### Uncertainty of frequency estimation

To properly estimate immunogenicity with frequency using formula (1), antigen/antibody frequency should be accurately assessed. However, frequency estimation may be challenging for low-/high-prevalence antigens. For low-prevalence antigens, antigen-positive frequency *p*_*x*_ for antigen x is low, showing a Poisson distribution. Additionally, the antibody frequency *Ab*_*x*_ for antigen x is low following a Poisson distribution. Therefore, if the antigen positivity/negativity, or antibody frequency, is *i* in a population of *n* people, each value shows a distribution of *Pois(i)*. In that case, according to the definition of Poisson distribution, the variance of the observed value is *i*, and the coefficient of variance (CV) is as follows:

10 $$ CV_{i} = \frac{SD}{{Mean}} = \frac{{\sqrt {Variance} }}{Mean} = \frac{\sqrt i }{i} $$

In formula (1), the effects of changes in the mean observed value *i* on total estimated antibody frequency were assessed.

Formula (1) was re-expressed as follows:

11 $$ Im_{x} = \frac{{Ab_{x} }}{{p_{x} \times q_{x} }} $$

Uncertainty propagation can be substituted to formula (11) as follows^10^:

12 $$ u^{2} \left( {Im_{x} } \right) = u^{2} \left( {Ab_{x} } \right) + u^{2} \left( {p_{x} } \right) + u^{2} \left( {q_{x} } \right) $$

## Results

### Expected antibody frequencies based on the basic formula

**Figure 1:**
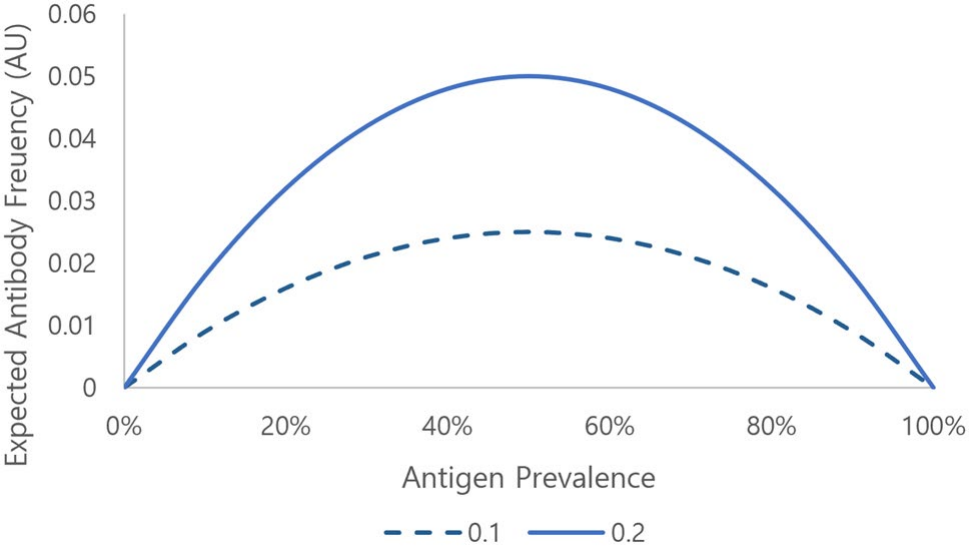
Expected antibody frequency according to antigen prevalence. Comparison of the results assuming immunogenicity as 0.1 and 0.2 is shown as an example.

Figure 1 shows the expected antibody frequency calculated with formula (1). As the antigen-positive rate was close to 0% or 100%, the expected antibody frequency was close to 0. This is consistent with intuitive estimation, indicating that antibody production is difficult because of the small number of antigen-positive blood units (~ 0%), or almost all recipients are antigen-positive (~ 100%). The graph shows that the expected antibody frequency increases as immunogenicity increases.

### Theoretical effect of multiple transfusions

**Figure 2:**
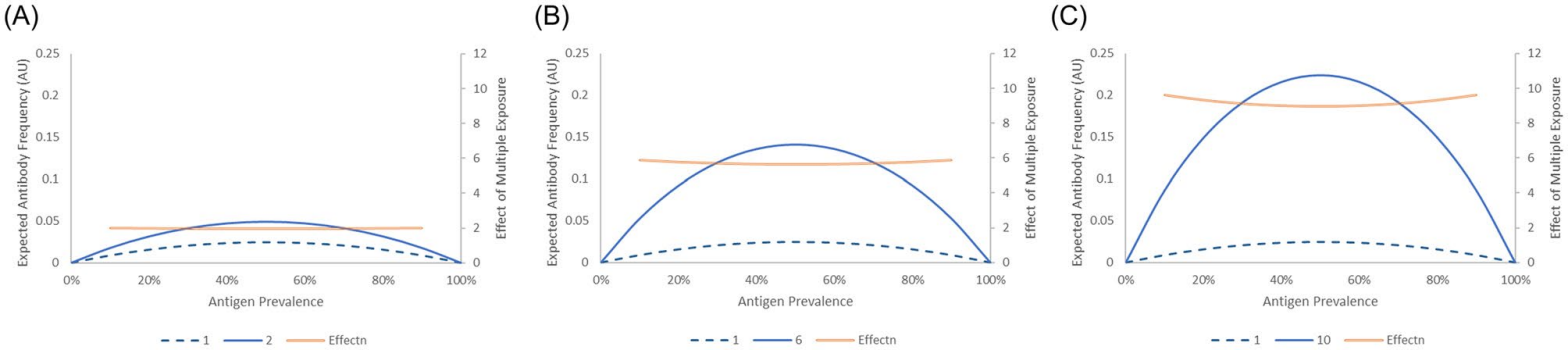
Effect of number of transfusion events on estimated antibody frequencies. As the number of events increased, the expected antibody frequency also increased. And the effects depend on the frequency of the antigen. The left Y-axis shows the estimated frequency of antibodies, while the right Y-axis displays the ratio of estimated antibody frequencies in multiple events compared to a single event (as shown by the dashed curve). (**A**) Effect of 2 transfusion events on estimated immunogenicity. (**B**) Effect of 6 transfusion events on estimated immunogenicity. (**C**) Effect of 10 transfusion events on estimated immunogenicity.

**Figure 3:**
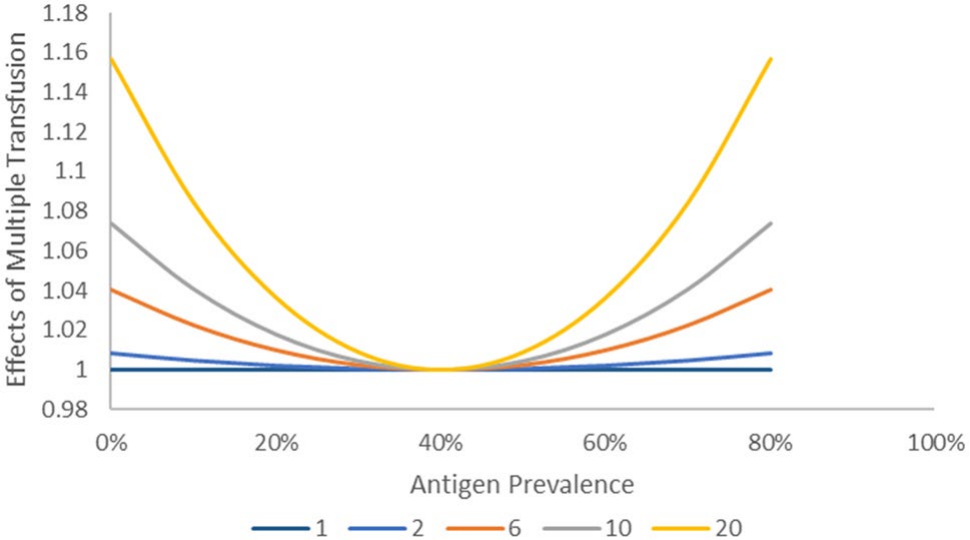
Ratio of multiple transfusion effects compared an antigen prevalence of 50%. The effect of multiple transfusion depends on the antigen prevalence.

Figure 2 shows the results of assuming multiple exposures using formulas (4) and (5). Similar to the graph in Fig. 1, the expected antibody frequency was close to 0 when the antigen prevalence was high or low. When multiple exposures were assumed, the expected antibody frequency tended to increase. To compare the expected antibody frequency calculated using formula (4) with that calculated using formula (1), the effect was calculated using formula (5). Value obtained using formula (4) tended to increase for high- or low-prevalence antigens (p ~ 0 or 1). This finding suggests that formula (1) may estimate falsely low immunogenicity for high-/low-prevalence antigens in populations with different antigen frequencies, even for antigens with the same immunogenicity. This effect tended to increase with increasing multiple exposures (Figs. 2 and 3).

### Theoretical effect of antigen density

**Figure 4:**
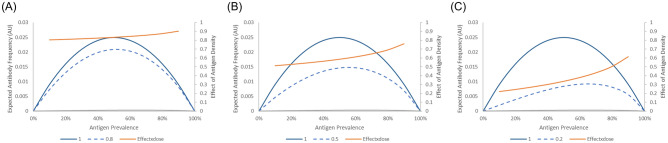
Effect of antigen density on estimated antibody frequencies. The left Y-axis shows the estimated frequency of antibodies, while the right Y-axis displays the ratio of estimated antibody frequencies when there is a difference in immunogenicity for heterozygote and homozygote cells. (**A**) Effect of antigen density on estimated antibody frequencies when the immunogenicity ratio is 0.8. (**B**) Effect of antigen density on estimated antibody frequencies when the immunogenicity ratio is 0.5. (**C**) Effect of antigen density on estimated antibody frequencies when the immunogenicity ratio is 0.2.

Figure 4 shows the theoretical effect of the antigen density on the expected antibody frequency using formula (9). Unlike the impact of multiple exposures, a low antigen positivity tended to significantly increase the effect of the antigen density. This suggests that formula (1) may lead to a falsely low estimation of immunogenicity. This is thought to reflect low-prevalence antigens in antigen-positive blood having a high chance of being a heterozygote. This effect increased as the effect of the antigen density increased.

### Uncertainty of frequency estimation

To evaluate the effects of the frequency estimation uncertainty on the immunogenicity estimation, values for antigen K from previous studies were used^6^ The antigen prevalence for K was obtained from Delaney et al.’s study, in which four out of 1,033 participants were positive for antigen K^11^ In addition, anti-K was detected in only ten out of 3,898 participants. Therefore, formula (10) was used to calculate CV. The antigen positivity, antigen negativity, and CV of the anti-K frequency were 50%, 1.6%, and 31.6%, respectively. Accordingly, the error propagation was used in formula (12), and the uncertainty of the estimated immunogenicity was high at 59.2%.

**Figure 5:**
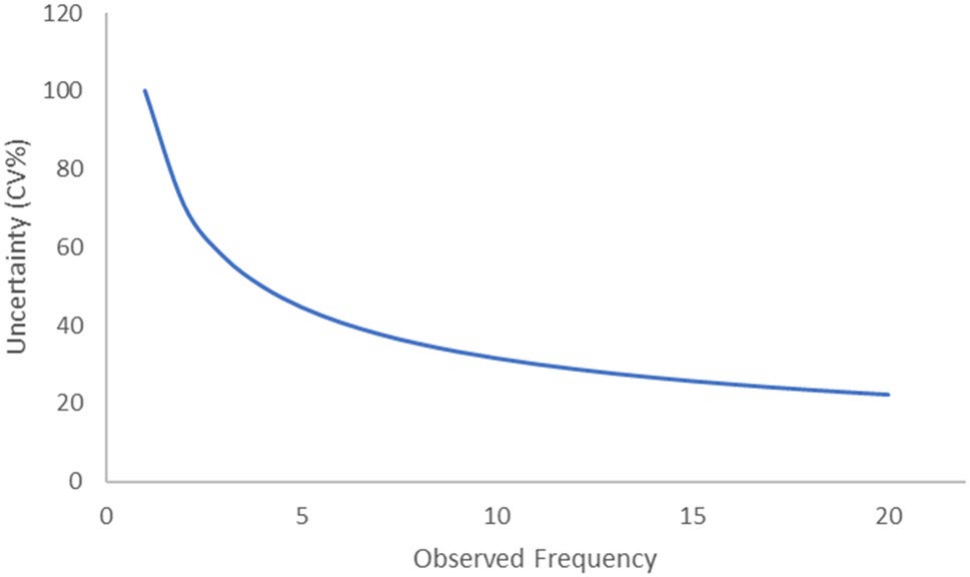
Relationship between number of observations and expected uncertainty of the observations. The uncertainties of frequencies less than 10 are higher than 30%.

Figure 5 shows the estimation of uncertainty using formula (10) and the observed frequency. The finding showed that estimation results for low frequencies should be interpreted with caution.

## Discussion

In this study, we investigated the effects of various factors potentially influencing the estimation of immunogenicity based on the antigen prevalence and antibody frequency. We demonstrated that multiple antigen exposures, dosage effects by antigen density, and uncertainty of low frequency may affect the estimated immunogenicity.

In particular, the level of effects of these factors on the antibody frequency varied with the antigen frequency in the population. Same antigens may show different allele distributions by race, which can lead to a false estimation of immunogenicity. In a previous study, the estimation of immunogenicity in Asians showed a relatively low immunogenicity of K and Fy(a), which are low-/high-prevalence antigens in Asians^6^ This finding is consistent with the results shown in Fig. 2.

Moreover, different populations have a different genetic background that affects immunologic reaction, which can ultimately influence immunogenicity. Immunogenicity may be influenced by human leukocyte antigen (HLA) molecules that are highly polymorphic and vary significantly among ethnic groups^12^ The peptide-binding affinity differs with the HLA type, resulting in variable immune responses by alloantigen exposure^13,14^.

In addition to the three factors discussed in this study, the antibody persistence period and condition of the transfused patient can also affect antibody frequency and, subsequently, the estimated immunogenicity. Due to the retrospective nature of the analysis, continued long-term presence of an antibody can increase the chance of the antibody frequency being estimated relatively high. These data can also be affected by the frequency of antibody testing according to the protocol at each institution. In a previous study, to exclude this effect, duplicate data of the same patient were not used, and only the initial data of each patient were analyzed^6^ However, this is not a fundamental solution to the problem. Moreover, the alloimmunization rate varies with the condition of the transfused patient even when the same incompatible blood is transfused^15^ These findings suggest that data from tertiary hospitals including many patients with cancer or history of transplantation would differ from the data of the general population.

Another factor not considered in the present or previous studies was allele variance. Each blood group antigen has variants. The frequency of these variant alleles is remarkably low such that they are not expected to impact the overall data significantly. However, if the allele frequency is low, similar to antigen K in Asians, the effect of the variant allele may be relatively significant because the antibody to the antigen is rarely discovered. The same is true for high-prevalence antigens. An example is the serologic RhD-negative antigen, which is rare, with a frequency of less than 1% in East Asians. Approximately 15% to 30% of serologic RhD-negative is Asia-type DEL (c.1227G > A), which has the same epitope as normal RhD and does not produce anti-D antibodies even when RhD-positive blood is transfused^16^ Estimating the immunogenicity of antigen D in the population using only the frequency of serologic RhD-negative and frequency of anti-D antibodies may significantly affect the results.

These findings reflect the inherent limitations of estimating immunogenicity retrospectively. The frequencies of pre- and post-transfusion tests may affect the antibody frequency. Furthermore, other variables, such as follow-up loss or a change of hospital, would limit the accuracy of the estimation of the total number of patients, unless a national registry is available.

As suggested in this study, estimating immunogenicity based on the antigen and antibody frequencies has limitations. In addition to blood transfusion, estimating immunogenicity based on the antibody frequency has been attempted for events in which antibodies are produced because of exposure to an alloantigen, such as in transplantation^17^ However, the same limitations would apply for such attempts as well.

There have been studies investigating the immunogenicity of blood group antigens from various perspectives. Some observations seem to contradict general expectations in terms of multiple exposures and antigen density. For instance, a study by Zalpuri et al. showed no significant difference in the occurrence of RBC alloimmunization between patients receiving intensive and non-intensive transfusions^18^ Additionally, Evers et al. reported a flattening of cumulative alloimmunization risk curves in some antigens, suggesting that the chance of alloimmunization diminishes with subsequent antigen exposure^19^ Arthur et al. demonstrated, using a mouse model, that low antigen density RBC-induced tolerance protects higher antigen density RBCs from immune-mediated clearance^20^ Moreover, Howe et al. reported that immunogenicity is positively related to total and ectodomain sizes of blood group proteins and negatively related to antigen site density based on glycosite predictions^21^ To accurately determine immunogenicity, well-designed prospective studies with minimal assumptions and formulas are needed. This study is meaningful as it shows the limitation of a widely used immunogenicity estimation formula by suggesting the various factors that may affect the calculation results.

Immunogenicity is used as evidence data to determine the antigens for extended antigen-matching. In patients with diseases such as thalassemia or sickle cell disease, extended antigen-matched transfusion is recommended for preventive purposes, as they frequently require multiple transfusions^3,22–24^ However, matching all known blood groups is impossible, and certain antigens should be selected for antigen-matching. This process should account for the clinical significance of alloantibodies, frequency of antigen-negative blood, and immunogenicity of the antigens. In other words, transfusion of antigens with low immunogenicity will have a relatively low probability of inducing alloimmunization in antigen-negative recipients. Thus, accurate estimation of immunogenicity for each antigen is essential; however, the lack of consistency in data from previous studies limits optimal evidence-based decision-making.

In conclusion, this study showed that immunogenicity estimation based on the antigen/antibody frequency in a certain population has limitations and is affected by variable factors. A well-designed prospective study with a large sample size is required to accurately estimate the immunogenicity of blood group antigens.

## Data Availability

The data that support the findings of this study are available on request from the corresponding author.
